# A molecular link between normal Pregnancy and Gestational diabetes mellitus in local population

**DOI:** 10.12669/pjms.41.6.12354

**Published:** 2025-06

**Authors:** Hafiz Muhammad Waseem, Huma Saeed Khan, Saba Khaliq, Lubna Javed

**Affiliations:** 1 Hafiz Muhammad Waseem, MBBS, M.Phil, PhD Assistant Professor, Department of Physiology, University of Health Sciences, Khyaban e Jamia, Lahore, Pakistan; 2 Huma Saeed Khan, MBBS, M.Phil, PhD Scholar Associate Professor, Department of Physiology, University of Health Sciences, Khyaban e Jamia, Lahore, Pakistan; 3 Saba Khaliq, M.Sc, PhD Director, Institute of Allied Health Sciences, University of Health Sciences, Khyaban e Jamia, Lahore, Pakistan; 4 Lubna Javed, MBBS, MCPS, FCPS Ex-Associate Professor, Department of Gynecology & Obstetrics, Allama Iqbal Medical College / Jinnah Hospital, Lahore, Pakistan

**Keywords:** Gestational diabetes mellitus, miR-16-5p, Insulin, IGF-1,TNF-α, Glutathione peroxidase, Biomarkers

## Abstract

**Objective:**

To assess the differential expression of miR-16-5p and associated biochemical markers in pregnancies complicated by gestational diabetes mellitus (GDM) compared to normal pregnancies.

**Methods:**

This cross-sectional comparative study included 80 pregnant women including 40 women with GDM and 40 age-matched healthy controls during their second and third trimesters. Participants were recruited from Lady Aitchison Hospital and Services Hospital, Lahore through random sampling from April 2021 to June 2022. Serum levels of insulin, insulin-like growth factor-1 (IGF-1), insulin-like growth factor binding protein-1 (IGFBP-1), tumor necrosis factor-alpha (TNF-α), and glutathione peroxidase (GPX) were measured using ELISA kits. miRNA was extracted from whole blood, and miR-16-5p expression was quantified using real-time polymerase chain reaction (PCR), with fold change calculated through the Livak method. Statistical analysis were conducted using IBM SPSS v26.0. Normality of the data was assessed with the Shapiro-Wilk test; Group comparisons were performed with the Mann-Whitney U test due to non-normal distribution of most variables, and associations of biochemical markers with miR-16-5p were evaluated using Spearman’s rank correlation.

**Results:**

GDM patients exhibited significantly higher serum glucose, insulin levels, and miR-16-5p expression (p < 0.001) compared to controls. Conversely, IGF-1, IGFBP-1, TNF-α, and GPX levels were elevated in the control group. In the GDM cohort, miR-16-5p expression positively correlated with glucose levels (r = 0.411, p = 0.008) and negatively with GPX levels (r = –0.450, p = 0.004).

**Conclusion:**

GDM-related pregnancies show significant differences in glucose, insulin, and TNF-α levels, with altered miRNA expression potentially serving as noninvasive biomarkers.

## INTRODUCTION

Gestational diabetes mellitus (GDM) is a transient hyperglycemia usually in the second half of pregnancy which is triggered by metabolic adaptations and resolves soon after parturition. Reported prevalence of GDM is highly variable based on diagnostic criteria, population demographics and study groups. Furthermore, increased incidence of GDM has been observed in recent years across the globe. The estimated global incidence of birth complications associated with hyperglycemia during pregnancy is approximately 16% annually.^1^ The overall pooled prevalence of GDM in Pakistan is 16.7%.^2^ The common etiological factors for GDM include family history of diabetes, advanced maternal age, obesity, and higher body mass index. Other reported risk factors include genetic makeup, polycystic ovarian syndrome (PCOS), ethnicity, and hypertension. Obesity, decreased insulin secretion, insulin resistance, oxidative stress, hormonal imbalance, and chronic inflammatory reaction have been associated in the pathogenesis of GDM.^3^

GDM mothers face an increased risk of developing Type 2 Diabetes Mellitus (T2DM), atherosclerosis, and obesity, while offspring may suffer from childhood obesity, overweight, macrosomia, shoulder dystocia, hyperinsulinemia, hypoglycemia, and hyper-bilirubinemia.^4^ Pre-natal complications include bacteriuria, pyelonephritis, pre-eclampsia, polyhydramnios, macrosomia, pre-term labor, stillbirths, and neonatal hypoglycemia.^5^ Early diagnosis and treatment of GDM can help prevent complications for both the mother and baby. GDM can alter circulating substances, potentially serving as diagnostic biomarkers. Researchers have explored various blood markers for gestational diabetes, including inflammatory markers, hormones, anti-oxidants, and proteins.^6^ miRNAs have been used as biomarkers for cancer, pre-eclampsia, hypertension, and diabetes, and are now being explored for novel biomarkers in GDM.^7^ Variable expression of 14 miRNAs was described by Villard including ten miRNAs with increased expression while remaining 4 having decreased expression in diabetic patients.^8^

Multiple studies have reported dysregulation of circulating miRNAs in diabetes. A meta-analysis of 38 studies revealed significant dysregulation of 40 miRNAs in diabetes, with eight proposed as potential biomarkers.^9^ In gestational diabetes mellitus (GDM), altered expression of specific miRNAs-such as increased miR-192 and miR-194 and decreased miR-29a, miR-132, and miR-222-has been observed. 10 According to literature, overexpression of miR-16-5p, miR-17-5p, and miR-20a-5p was consistently reported in GDM patients.^11^

Given the high variability in the results, diverse ethnicity and unavailability of the literature on utility of miRNA in GDM in Pakistani population, the present study was designed to identify fold change in miRNA expression (miR-16-5p) in GDM patients and control subjects, compare glucose, insulin, insulin-like growth factor-1, glutathione peroxidase, and TNF-α levels, and correlate these biochemical parameters with miRNA expression.

## METHODS

This was a cross-sectional comparative study comprising of 80 pregnant females including 40 GDM and 40 age-matched controls during 2^nd^ and 3^rd^ trimester of pregnancy. The subjects were recruited through random sampling from Lady Aitchison Hospital, Lahore and Services Hospital, Lahore from April 2021 to June 2022.

### Ethical Approval:

The study was approved by ethical review board of University of Health Sciences vide letter No. UHS/REG-19/ERC/2305 dated 28^th^ June, 2019.

### Inclusion criteria:

Pregnant women aged 18–40 years with pregnancies at 24–28 weeks of gestation were enrolled after providing informed consent. The GDM group included participants diagnosed according to ADA/IADPSG criteria via a 75g OGTT, while the control group comprised women with normal glucose tolerance.

### Exclusion criteria:

Females having any chronic illness e.g. chronic kidney disease, liver disease, hypertension, history of infection and use of glucose-altering medications in previous 2-4 weeks or any chronic inflammatory disease were excluded from the study.

For the subjects fulfilling eligibility criteria, OGTT was performed using one step approach as described by American Diabetes Association^12^ if the subject was not already tested for GDM using the same method. Around 5ml blood was then drawn under aseptic measures. Two ml blood was added into vacutainer® tube containing Na-citrate as an anti-coagulant to be used later for extraction of miRNAs while 3 ml blood was added into vacutainer® tube containing clot activator for separation of serum to be used for measurement of blood biomarkers. The samples were processed at Physiology Laboratory, University of Health Sciences, Lahore.

Serum Human Insulin Level was measured using commercially available ELISA kit manufactured by Calbiotech, 1935 Cordell Court, El Cajon, CA 92020 while serum levels of remaining blood biomarkers [Human insulin-like growth factor-1(IGF-1), human insulin-like growth factor binding protein-1 (IGFBP-1), human tumor necrosis factor-alpha (TNF-α) and human glutathione peroxidase (GPX)] were measured using commercially available ELISA kits manufactured by Bioassay Technology Laboratory, 228 Ningguo Rd, Yangpu Dist 200090, Shanghai, China. MiRNA was extracted from whole blood using Invirogen™ mirVana™ miRNA Isolation Kits manufactured by Thermo Fisher Scientific, 168 Third Avenue Waltham, MA 02451 USA. cDNA was then synthesized from miRNA according to manufacturer’s protocol using RevertAid First Strand cDNA Synthesis Kit by Thermo Scientific, 168 Third Avenue Waltham, MA 02451 USA.

Expression of miR-16-5p was detected on CFX96 Real Time PCR detection system using Maxima SYBR Green by Thermo Scientific, USA. Experiment for relative expression of miRNA was performed with biological and technical replicates with U-6 as housekeeping gene. Fold change of miRNA was determined using Livak method.^13^ Statistical Package for Social Sciences (SPSS) version 26.0 was used for data entry and analysis. Median (IQR) values were used for quantitative variables. Mann Whitney U Rank Sum test was used to compare various biochemical parameters while Spearman Rho correlation analysis was performed to determine the correlation between miR-16-5p and biochemical parameters.

## RESULTS

A total of N=80 subjects were recruited for the study. The data of all 80 subjects was explored for normality by using the Shapiro-Wilk Test as shown in Table-I. The variables with statistically significant p-value were considered to be non-normally distributed. The median (IQR) age of the study participants was 26.00 (24.25-30.00) years. The median (IQR) gestational age at the time of inclusion into the study was 34.20 (29.10-36.40) weeks (Table-I). Serum insulin was found higher in the GDM group while levels of IGF-1, IGFBP-1, tumor necrosis factor-alpha, and glutathione peroxidase were higher in the control group.

**Table-I:** Age and biochemical parameters (N=80).

Study parameters	n=80 Mean ± SD	n=80 Median (IQR)	Distribution (p-value)
Age (years)	26.81 ± 4.35	26.00 (24.25 – 30.00)	0.014*
Gestational age (weeks)	32.90 ± 4.31	34.20 (29.10 – 36.40)	<0.001*
***Biochemical parameters (n=80)***			
Serum glucose level (mg/dl)	108.24 ± 39.05	94.00 (88.00 – 116.75)	<0.001*
Human insulin level (pmol/L)	45.45 ± 59.50	23.13 (13.69 – 46.22)	<0.001*
Human insulin-like growth factor-1 level (ng/ml)	42.55 ± 5.52	43.68 (41.89 – 45.07)	<0.001*
Human Insulin-like growth factor binding protein-1 Level (ng/ml)	298.87 ± 37.03	298.78 (284.69 – 314.55)	<0.001*
Human tumor necrosis factor-alpha– level (ng/L)	81.23 ± 27.33	76.14 (66.42 – 86.29)	<0.001*
Human glutathione peroxidase level (mg/ml)	17.51 ± 5.10	16.53 (14.93 – 19.13)	<0.001*
miR-16-5p (fc)	1.41 ± 0.57	1.27 (0.95 – 1.63)	<0.001*

* Variables with p-value <0.001 are non-normally distributed (Shapiro-Wilk Test).

Mann Whitney U Rank Sum test was used to compare various biochemical parameters in the study population irrespective of gestational age. Statistically significant differences were observed between the groups in serum glucose level, human insulin levels and fold change in miR-16-5p (Table-II). Spearman’s rank correlation was performed to evaluate the association between miR-16-5p and the biochemical markers in both GDM and control groups (Table-III). In the GDM group, miR-16-5p showed a significant positive correlation with serum glucose (r = 0.360, p = 0.023) and a negative correlation with glutathione peroxidase (r = –0.450, p = 0.004). No statistically significant correlations were observed in the control group.

**Table-II:** Comparison of various biochemical parameters and miR-16-5p in GDM and Control.

Study Parameter	Groups	n	Rank Average	Sum of Ranks	U	Z	P
Serum glucose level	GDM	40	52.31	2092.50	327.50	1147.50	<0.001*
	Control	40	28.69	1147.50			
Human Insulin Level	GDM	40	48.78	1951.00	469.00	-3.185	0.001*
	Control	40	32.23	1289.00			
Human Insulin-Like growth Factor Level	GDM	40	35.80	1432.00	612.00	-1.809	0.070
	Control	40	45.20	1808.00			
Human IGFBP-1 level	GDM	40	40.23	1609.00	789.00	-0.106	0.916
	Control	40	40.78	1631.00			
Human Tumor Necrosis Factor - Level	GDM	40	37.14	1485.50	665.50	-1.294	0.196
	Control	40	43.86	1754.50			
Human Glutathione Peroxidase Level	GDM	40	37.44	1497.50	677.50	-1.179	0.238
	Control	40	43.56	1742.50			
miR-16-5p (fc)	GDM	40	58.00	2320.00	100.00	-6.737	<0.001*
	Control	40	23.00	920.00			

* p≤0.05 is considered statistically significant

**Table-III:** Spearman’s Correlation Between miR-16-5p and Biochemical Parameters in GDM and Control Groups.

Parameter	GDM		Control	
	Correlation coefficient (r) n=40	p-value	Correlation coefficient (r) n=40	p-value
Serum Glucose level	0.360	0.023*	0.037	0.823
Serum human Insulin Level	-0.037	0.820	0.060	0.714
Human Insulin-like growth factor-1 Level	0.020	0.902	0.047	0.771
Human Insulin-like growth factor binding protein -1 Level	-0.093	0.567	-0.158	0.329
Human Glutathione peroxidase Level	-0.450	0.004*	-0.022	0.892
Human Tumor necrosis factor	-0.267	0.096	-0.053	0.748

* p≤0.05 is considered statistically significant

## DISCUSSION

Women with gestational diabetes mellitus (GDM) are at higher risk of GDM recurrence and developing Type-2 diabetes later in life, with their offspring prone to metabolic complications. Emerging evidence highlights microRNAs (miRNAs) as early biomarkers involved in GDM pathogenesis by regulating glucose homeostasis, insulin sensitivity, and inflammation, offering potential for early diagnosis and intervention.^14^ Juchnicka et al.^15^ emphasized that miRNA have a definite predictive role in the pathophysiology and early diagnosis of GDM. They further validated miR-16-5p, miR-142-3p, miR-144-3p, and miR-320 having prominent changes between GDM and control groups.

The current study aimed to determine association between altered expression of miR-16-5p and its association with TNF-α, GPX level and hormones in GDM patients compared to healthy controls. The results of the current study indicated upregulation of miR-16-5p in GDM subjects as compared to healthy controls. The levels steadily increased with increase in gestation age. Similar to our results, miR-16-5p is the most frequently up-regulated miRNA in different studies on GDM.^16^,^17^ Analysis of these studies further reveals that the raised levels of these miRNAs are positively correlated with insulin resistance and remain increased during the remaining two trimesters of pregnancy.^15^ Other studies have associated over-expression of miR-16-5p with a concomitant decrease in levels of IRS-1 and IRS-2 leading to insulin resistance.^18^,^19^ MiR-16 targets genes involved in insulin signaling, potentially causing insulin resistance and cell apoptosis. In the current study, no correlation was observed between miR-16 levels and serum IGF-1 and IGFBP-1 levels. The difference from the current study and other studies could probably be due to genetic and ethnic differences between study subjects, diagnostic criteria used for identification of GDM, duration of pregnancy, family history, co-morbidities and environmental factors.

Oxidative stress in GDM women leads to increased blood glucose concentration, generating free radicals and depleting antioxidants. MiR-16-5p reduces expression of GPX, potentially promoting oxidative stress in the cell. In current study, GPX levels were lower in GDM patients as compared to the controls. Similar results have been reported by Zhang et al.^20^ in a case-control study in Chinese Population. The marked increase in oxidative stress in later trimesters can be related to striking derangement in glucose levels. Furthermore, use of naturally occurring anti-oxidants as well as anti-oxidant therapy which protects the beta-cell against oxidative stress-induced apoptosis, preserves beta-cell function, and reduces diabetic-related complications.^21^

MicroRNA-16 has been found to have anti-inflammatory effects by reducing pro-inflammatory factors like IL-6, TNF-α, and MCP-1 while promoting anti-inflammatory factors like IL-10 and TGF-β. Transfection with miR-16 suppressed the secretion and mRNA expression of pro-inflammatory factors, such as IL-6 and TNF-α.^22^ This is mediated by downregulating targets like NF κB and NLRP3 inflammasome.^23^ A review of literature indicates that TNF-α and IL-6 are the major inflammatory cytokines involved in disruption of insulin signaling and insulin resistance in GDM and are positively correlated in gestational diabetes.^24^ Similar results were seen in the current study, where TNF- α level were lower in GDM group. Studies have reported raised median TNF- α level^25^ as well its decreased level^26^ in GDM patients. Still other studies have reported no effect of TNF-α gene polymorphism on increasing risk of GDM.^27^ While many studies have reported significant association between elevated levels of TNF-α levels and GDM, a number of studies have failed to identify any such association in pregnant women with GDM.

Understanding the precise role of miRNAs could help early detection of GDM pregnancies and potentially prevent the short and long-term complication both in mother and fetus. Furthermore, this could help in the development of more targeted and effective therapeutic interventions for GDM. Therefore, future researches in understanding the functions and implications of miRNAs in the context of GDM remains critical for better clinical outcome.

### Limitations and Future recommendations:

The study’s major limitation is its cross-sectional design, which may not consider factors like fetus gender and pregnancies affecting miRNA expression in maternal blood. Next-generation sequencing could identify and validate over and under-expressed miRNAs in GDM patients, with further prospective cohort studies to investigate factors affecting miRNA expression.

## CONCLUSION

Serum levels of glucose, insulin, insulin-like growth factor-1, glutathione peroxidase, and TNF-α were significantly different in pregnancies complicated with GDM compared to healthy subjects. Differential expression of miRNAs was observed in GDM patients as compared to controls. Altered expression of miR-16-5p was positively correlated with glucose level and negatively correlated with glutathione peroxidase levels. It was concluded that altered expression of miR-16-5p in women with GDM may serve as noninvasive biomarkers and could be used for the identification of underlying mechanisms for gestational diabetes and pregnancy-related complications.

### Author’s Contribution:

**HMW:** Concept design, acquisition of data and writing of manuscript.

**HSK:** Literature search, Statistical analysis and Critical Review.

**SK:** Acquisition of data, Manuscript review and Critical analysis.

**LJ:** Concept design, Critical Review

All authors have read and approved the final version. They are also responsible for integrity of research.

## References

[1] Riaz M, Waris N, Saadat A, Fawwad A, Basit A (2024). Gestational diabetes mellitus as a risk factor for future Type-2 diabetes mellitus:An experience from a tertiary care diabetes hospital, Karachi-Pakistan. Pak J Med Sci.

[2] Adnan M, Aasim M (2024). Prevalence of gestational diabetes mellitus in Pakistan:a systematic review and meta-analysis. BMC Pregnancy Childbirth.

[3] Ouyang P, Duan S, You Y, Jia X, Yang L (2023). Risk prediction of gestational diabetes mellitus in women with polycystic ovary syndrome based on a nomogram model. BMC Pregnancy Childbirth.

[4] Parveen N, Iqbal N, Batool A, Mahmoud T, Ali S (2022). Macrosomia predictors and pregnancy outcomes in Gestational Diabetes patients:An observational study from Ha'il, Saudi Arabia. Pak J Med Sci.

[5] Poirier C, Desgagne V, Guerin R, Bouchard L (2017). MicroRNAs in pregnancy and gestational diabetes mellitus:Emerging role in maternal metabolic regulation. Curr Diab Rep.

[6] Di Filippo D, Wanniarachchi T, Wei D, Yang JJ, Mc Sweeney AM, Havard A (2021). The diagnostic indicators of gestational diabetes mellitus from second trimester to birth:a systematic review. Clin Diabetes Endocrinol.

[7] Karami M, Mousavi SH, Rafiee M, Heidari R, Shahrokhi SZ (2023). Biochemical and molecular biomarkers:unraveling their role in gestational diabetes mellitus. Diabetol Metab Syndr.

[8] Villard A, Marchand L, Thivolet C, Rome S (2015). Diagnostic value of cell-free circulating micrornas for obesity and type 2 diabetes:A meta-analysis. J Mol Biomark Diagn.

[9] Zhu Y, Tian F, Li H, Zhou Y, Lu J, Ge Q (2015). Profiling maternal plasma microRNA expression in early pregnancy to predict gestational diabetes mellitus. Int J Gynaecol Obstet.

[10] Cao YL, Jia YJ, Xing BH, Shi DD, Dong XJ (2017). Plasma microRNA-16-5p, -17-5p and -20a-5p:Novel diagnostic biomarkers for gestational diabetes mellitus. J Obstet Gynaecol Res.

[11] Balcı S, Gorur A, Derici Yıldırım D, Çayan F, Tamer L (2020). Expression level of miRNAS in patients with gestational diabetes. Turk J Bichem.

[12] American Diabetes Association (2018). 2 Classification and diagnosis of diabetes:Standards of medical care in diabetes-2018. Diabetes Care.

[13] Livak KJ, Schmittgen TD (2001). Analysis of relative gene expression data using real-time quantitative PCR and the 2-ΔΔCT method. Methods.

[14] Elhag DA, Al Khodor S (2023). Exploring the potential of microRNA as a diagnostic tool for gestational diabetes. Transl Med.

[15] Juchnicka I, Kuzmicki M, Niemira M, Bielska A, Sidorkiewicz I, Zbucka-Kretowska M (2022). miRNAs as predictive factors in early diagnosis of gestational diabetes mellitus. Front Endocrinol (Lausanne).

[16] Filardi T, Catanzaro G, Grieco GE, Splendiani E, Trocchianesi S, Santangelo C (2022). Identification and validation of miR-222-3p and miR-409-3p as plasma biomarkers in gestational diabetes mellitus sharing validated target genes involved in metabolic homeostasis. Int J Mol Sci.

[17] Sorensen AE, Van Poppel MNM, Desoye G, Damm P, Simmons D, Jensen DM (2021). The predictive value of miR-16, -29a and -134 for early identification of gestational diabetes:A nested analysis of the DALI cohort. Cells.

[18] Kunysz M, Cieśla M, Darmochwał-Kolarz D (2024). Evaluation of miRNA expression in patients with gestational diabetes mellitus:Investigating diagnostic potential and clinical implications. Diabetes Metab Syndr Obes.

[19] Li J, Gan B, Lu L, Chen L, Yan J (2023). Expression of microRNAs in patients with gestational diabetes mellitus:a systematic review and meta-analysis. Acta Diabetol.

[20] Zhang B, Zhang T, Hu S, Sun L (2022). Association of serum lipid peroxidation and glutathione peroxidase 4 levels with clinical outcomes and metabolic abnormalities among patients with gestational diabetes mellitus:a case-control study in the Chinese population. FBL.

[21] Shafras M, Sabaragamuwa R, Suwair M (2024). Role of dietary antioxidants in diabetes:An overview. Food Chem Adv.

[22] Liang X, Xu Z, Yuan M, Zhang Y, Zhao B, Wang J (2016). MicroRNA-16 suppresses the activation of inflammatory macrophages in atherosclerosis by targeting PDCD4. Int J Mol Med.

[23] Yang M, Peng S, Li W, Wan Z, Fan L, Du Y (2016). Relationships between plasma leptin levels, leptin G2548A, leptin receptor Gln223Arg polymorphisms and gestational diabetes mellitus in Chinese population. Sci Rep.

[24] Omazic J, Viljetic B, Ivic V, Kadivnik M, Zibar L, Muller A (2021). Early markers of gestational diabetes mellitus:what we know and which way forward?. Biochem Med (Zagreb).

[25] Chiti H, Izadi MH, Mazloomzadeh S (2020). The association between pregnancy serum TNF-αLevel and postpartum insulin resistance in pregnant women with gestational diabetes mellitus. Acta Medica Iranica.

[26] Abdullahi M, Ibrahim SA (2018). Maternal serum level of TNF-&in Nigerian women with gestational diabetes mellitus. Pan Afr Med J.

[27] Huang Q, Wang Y, Gu B, Xu Y (2020). Whether the risk of gestational diabetes mellitus is affected by TNF-α, IL-6, IL-10 or ADIPOQ polymorphisms:a meta-analysis. Diabetol Metab Syndr.

